# Natural killer cell activity in metastatic castration resistant prostate cancer patients treated with enzalutamide

**DOI:** 10.1038/s41598-023-43937-7

**Published:** 2023-10-10

**Authors:** A. H. Zedan, L. Nederby, L. M. Volmer, C. V. Madsen, B. E. Sørensen, T. F. Hansen

**Affiliations:** 1 Department of Oncology, Vejle Hospital, University Hospital of Southern Denmark, Vejle, Denmark; 2 Department of Biochemistry and Immunology, Vejle Hospital, University Hospital of Southern Denmark, Vejle, Denmark; 3 Institute of Regional Health Research, University of Southern Denmark, Odense, Denmark

**Keywords:** Prostate cancer, Tumour immunology

## Abstract

Metastatic castration resistant prostate cancer (mCRPC) is still the lethal stage for the whole spectrum of prostate cancer disease. Even though different treatment options have been introduced in the last decade with a significant survival improvement for this population, a lack of more reliable prognostic and predictive markers is still one of the main clinical challenges in management of mCRPC. The aim of this study was to investigate the correlation between Natural Killer cell activity (NKA) and both treatment effect and outcomes in patients with mCRPC treated with enzalutamide. A total of 87 patients with mCRPC treated with enzalutamide as the first line treatment were enrolled. NKA was estimated at baseline and prior to each treatment cycle. Endpoints included both treatment effect with biochemical response (BR), biochemical progression (BP) and radiological progression (RP), as well as outcome data with overall survival (OS), radiologic progression free survival (rPFS), and time to next treatment (TTT). At the time of BR, interferon-gamma (IFNγ) decreased significantly compared to levels detected at baseline (z-score = 2.33, p = 0.019). Regarding outcome data, the whole cohort was divided into four groups according to the change of IFNγ level during the first 3 cycles of enzalutamide treatment. In group 1 (n = 42) the IFNγ level remained within a normal range (≥ 250 pg/mL),while in group 2 (n = 7) it increased from an abnormal (< 250 pg/mL) to a normal level. In group 3 (n = 13) it dropped to an abnormal level, and it remained at an abnormal level during treatment in group 4 (n = 17). Patients in group 2 showed the worst prognosis with shorter both rPFS and TTT (HR 4.30, p = 0.037; and HR 6.82, p = 0.011, respectively). In this study inverse correlations between NKA and both treatment response and outcomes was observed in mCRPC patients receiving enzalutamide, suggesting an unfavourable role of NK cells in the late stage of PCa.

## Introduction

The management of metastatic prostate cancer (mPC) has improved markedly in the last decade with many different treatment modalities resulting in an obvious survival benefit up to several years^1^.

Changes in treatment sequences, combination strategies and development of new management options rather than traditional chemotherapy, such as new anti-hormonal agents, vaccine therapy, radioactive targeted therapy, and immunotherapy has noticeably lead to such benefit. However, metastatic castration resistant prostate cancer (mCRPC) is still the lethal stage of the whole prostate cancer (PC) spectrum with an overall survival (OS) that barely reaches 3 years^2^.

Enzalutamide (formerly known as MDV3100) is one of the new androgen-receptor targeted treatments that competitively binds to the androgen receptor and inhibits its translocation at several levels^3^. It has been approved as one of the effective androgen deprivation therapies (ADT) for both de novo metastatic castration sensitive PC (mCSPC)^4,5^ and for CRPC with^6,7^ and without metastases^8^.

Since early 1990s, more than fifty prognostic models have been developed for the mCRPC population in an attempt to guide clinicians during the decision-making process, but most of these models are still challenged by many pitfalls such as validation, interpretation and reliability^9^. Different prognostic factors are widely explored in these models including neutrophil to lymphocyte ratio (NLR), as there has been an accumulating evidence suggesting that host inflammation response is closely associated with both tumorigenesis and tumor progression^10^.

Although they were discovered for about half a century ago^11,12^, natural killer (NK) cells have only more recently been attracting special attention for their critical role in tumor immuno-surveillance. Natural Killer cells account for about 10% of all peripheral blood lymphocytes, and play a pivotal role in both innate and adaptive immune responses against not only viral infection but also tumor development^13^. One of the crucial functions of NK cells is the release of cytokines with both tumoricidal and chemoattractant properties. By means of density of the cell surface receptor CD56 NK cells can be divided into two main subsets: The CD56^dim^ subset, that constitute the majority of the circulating NK cells and has high cytotoxic potential, and the CD56^bright^ subset which produce large amounts of various cytokines and chemokines^14,15^.

Interferon-γ (IFNγ) is a cytokine known to impact tumorigenesis. It is predominantly secreted by activated CD4 + T-cells, CD8 + T-cells, NK T-cells and NK-cells, while antigen presenting cells such as B-cells and monocytes also produce IFNγ^16,17^. Besides activating cellular immune responses against malignant cells, IFNγ has also anti-proliferate, pro-apoptotic and antitumor effects^16^. Although IFNγ is most often positively associated with better clinical outcome in cancer patients there are indications that this cytokine has a pro-tumorigenic role as well^17^. The tumor modulation role of IFNγ has been widely elucidated in diverse types of both hematological and solid malignancies including PC^18^.

In this study, we explored the possible correlation between NK cell activity (NKA) as measured by the NK Vue® assay and both treatment response and outcome in patients diagnosed with mCRPC and treated with enzalutamide.

## Material and methods

### Patients

This study was based on blood samples prospectively collected from mCRPC patients who received enzalutamide as first-line treatment in mCRPC stage. The patients were enrolled at the Department of Oncology, Vejle Hospital, Denmark. The study was approved by The Regional Committees on Health Research Ethics for Southern Denmark (S-20160029) and by The Danish Data Protection Agency according to Danish law. The Danish Registry of Tissue Utilization was screened prior to analysis initiation. All methods were performed in accordance with the relevant guidelines and regulations.

**Figure 1:**
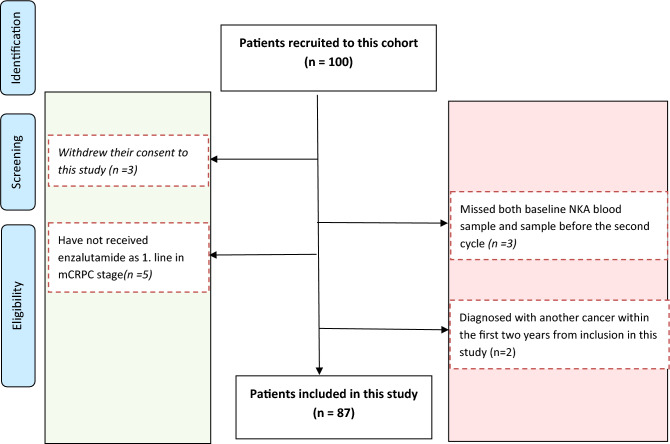
PRISMA flow chart.

In total, 100 mCRPC patients were recruited from October 2016 to September 2020. However only 87 patients were included in this study, as thirteen patients were excluded; three patients withdrew their consent, five patients did not receive enzalutamide as first line treatment, three patients missed the NKA evaluation both at baseline and before the second cycle of therapy, and two patients were diagnosed with another type of cancer within a period of less than two years from first cycle of enzalutamide. All participants in this study have provided informed written consent. Figure 1 shows PRISMA flow chart of both inclusion and selection of these patients to the current study.

Patients received enzalutamide tablets 160 mg once daily until radiological progression or unacceptable toxicity. In some patients, the total dose was reduced to either 120 or 80 mg daily due to intolerable side effects.

All patients were evaluated biochemically with 4-weeks interval, clinically every 8 weeks, and radiologically every 16 weeks; for bone metastases by either bone scintigraphy or 18F Sodium Fluoride positron emission tomography (NaF/PET) scan as well as chest and abdomen computed tomography (CT) for soft tissue metastases.

The follow-up period was defined as the time from first cycle (one cycle is about 28 days) of enzalutamide to November 11, 2021. Time to mCRPC was calculated from the date of PC diagnosis to the date of verified mCRPC. The definition of mCRPC was based on the European Association of Urology (EAU) guidelines^19^. Biochemical response (BR), biochemical progression (BP) and radiological progression (RP) were assessed on the basis of the Prostate Cancer Working Group 3 criteria^20^, while soft-tissue disease progression was evaluated by modified Response Evaluation Criteria In Solid Tumors (RECIST) 1.1^21^.

Radiologic progression free survival (rPFS) was the time from treatment start to radiological progression or death, whichever occurred first, and OS was defined as the time from treatment start to death from any cause. Time to new treatment (TTT) was defined as the time from first cycle of enzalutamide to the time of initiation of new treatment for mCRPC regardless the reason.

### Sampling and NK cell activity analyses

Peripheral blood was sampled before initiation of treatment (baseline) and prior to each treatment cycle until RP or treatment change due to any causes other than RP.NKA was measured indirectly using the NK Vue® assay. The principle of this test has previously been accounted for by our group^22^. Specifically, 1 mL of venous blood was drawn from the antecubital area into an NK Vue® tube (NKMAX, Seongnam-si, South Korea) and placed at 37 °C within 15 min of sampling. After 20–24 h the plasma was collected and stored at − 80 °C until analysis. Subsequently, the levels of IFNγ in thawed plasma samples were measured as a surrogate marker of NKA. Interferon gamma was assessed by the NK Vue® ELISA (NKMAX) according to manufacturer’s recommendations. Samples with levels of IFNγ above the upper limit of the assay (2000 pg/mL) were reanalyzed in a 1:10 dilution. Samples having IFNγ levels below the limit of quantification (65 pg/mL) were given a fixed value of 32 pg/mL. The in-house intra-assay coefficient of variation of the ELISA was < 10% while the inter-assay coefficient of variation was < 12%. According to the manufacturer’s instructions, a cutoff of 250 pg/mL may be used to distinguish between abnormal (< 250 pg/mL) and normal (≥ 250 pg/mL) values of NKA. Sampling and analyses were performed by staff unaware of patient outcome.

### Statistics

The Wilcoxon signed rank sum test was used to test for differences between plasma level of IFNγ at baseline, after one treatment cycle, and at BR, BP and RP.

The assessment of the differences in baseline clinical characteristics between patients in the four groups, an Equality of Medians test was used for numerical variables and Chi2 test was used for categorical variable, while Wilcoxon rank sum test was applied to assess the differences between patients in group 2 and the rest of the cohort.

Plasma level of IFNγ were tested for correlation with baseline clinical characteristics at mCRPC using Spearman's rank correlation.

The association between IFNγ and OS and rPFS was assessed using simple Cox regression and Kaplan–Meier survival curves. Proportional hazard assumption for the Cox regression model was assessed by Schoenfeldt residuals, and log-rank was used to test for differences between Kaplan Meier survival curves. Cox regression was adjusted for both the International Society of Urological Pathology (ISUP) grade, baseline Neutrophil, and NLR.

All analyses were performed in Stata version 16.1 (StataCorp LLC, TX, USA), and correlations/differences were considered statistically significant when a p-value was < 0.05.

## Results

The mean age at inclusion was 76 years (54–89 years), and median baseline prostate specific antigen (PSA) level at the stage of mCRPC was 50 ng/mL. About 95% of patients had a good performance status (0–1) at time of inclusion. Half of the patients had a high ISUP grade (≥ IV). More than half of this cohort (57%) had de novo mCSPC, of whom 30% had received docetaxel up-front. Previous treatment by docetaxel had no significant effect on NKA in the first three cycles of enzalutamide treatment (data not shown). About 17% of the whole population had received curatively intended therapy as either radiotherapy (RT) or surgery. The clinic-pathological characteristics of all patients is presented in Table 1

**Table 1:** Baseline clinic-pathological characteristics.

Mean age, years (range)	76 (54–89)
PS	
0	49
1	24
2	4
N/A	10
Pathology	
Adenocarcinoma	87
ISUP	
I	8
II	7
III	18
IV	19
V	33
N/A	2
Type of initial management	
RARP	6
RT	9
DOC upfront	15
Time from initial management to 1. cycle enzalutamide, mo, median (range)	25 (4–231)
RARP	135 (18–231)
RT	89 (26–168)
DOC upfront	17 (4–25)
Type of castration treatment	
Medical	81
Medical then surgical	6
Median PSA level, ng/mL (range)	50 (1–396)
Site of metastases*	
Bone	73
LN	39
Lung	5
Others	3
Time to mCRPC, mo, median (range)	68 (8–238)
Follow-up, mo, median (range)	39 (14–62)
Time to BP, mo, median (range)	7 (1–47)
Time to RP, mo, median (range)	20 (2–53)
Death	42
Synchronic cancer	2
Previous Cancer	7
Auto-immune disease	6

*ADT* anti-deprivation therapy, *BP* biochemical progression, *DOC* docetaxel, Dx diagnosis, *ISUP* International Society of Urological Pathology, *LN* lymph node, *mCRPC* metastatic castration resistant prostate cancer, *mo* month, *PSA* prostate specific antigen, *PS* performance status, *RARP* robot assisted radical prostatectomy, *RP* radiological progression, *RT* radiotherapy.

*Some patients have metastases to more than one site.

### Changes in NKA during treatment

Plasma levels of IFNγ at baseline was compared to values measured after one cycle of enzalutamide, at BR, at BP and at time of RP (Fig. 1). The analyses showed that the level of IFNγ was significantly lower at the time of BR compared to baseline levels (z = − 2.33, p = 0.019), while the remaining did not reach statistical significance (Table 2). Furthermore, when comparing levels of IFNγ at BR, BP, and RP there was a significant decrease in IFNγ at RP compared to BP (z = − 2.17, p = 0.029).

**Table 2:** Changes in plasma level of IFNγ during treatment with Enzalutamide:

Sample	z-score	p-value
Baseline vs. 1. cycle	1.78	0.075
Baseline vs. BR	2.33	**0.019**
Baseline vs. BP	0.44	0.671
Baseline vs. RP	1.57	0.120
BR vs. BP	− 0.95	0.351
BR vs. RP	0.12	0.913
BP vs. RP	2.17	**0.029**

Statistically significant values are in bold writing.

*BR* biochemical response, *BP* biochemical progression, *RP* radiological progression.

### Association between NKA and treatment outcome

The cohort was divided into four groups based on changes in plasma level of IFNγ during the first 3 cycles of enzalutamide treatment. In total, 79 patients made up all four groups, as eight more patients were excluded in this analysis due to missing at least two NKA evaluations during the first three cycles of the measurement. In group 1 (n = 42), plasma level of IFNγ remained within a normal range (≥ 250 pg/mL), while patients in group 2 (n = 7) were characterized by an increase of IFNγ from an abnormal level at baseline (< 250 pg/mL) to normal values. In group 3, (n = 13) levels of plasma IFNγ dropped to an abnormal level, whereas patients in group 4 remained at an abnormal level (n = 17). 

Baseline clinical characteristics for patients in these four groups are summarized in Table S1, and levels of IFNγ in the first three cycles of enzalutamide treatments for these four groups are also illustrated in both Figs. S1 and S2.

**Figure 2:**
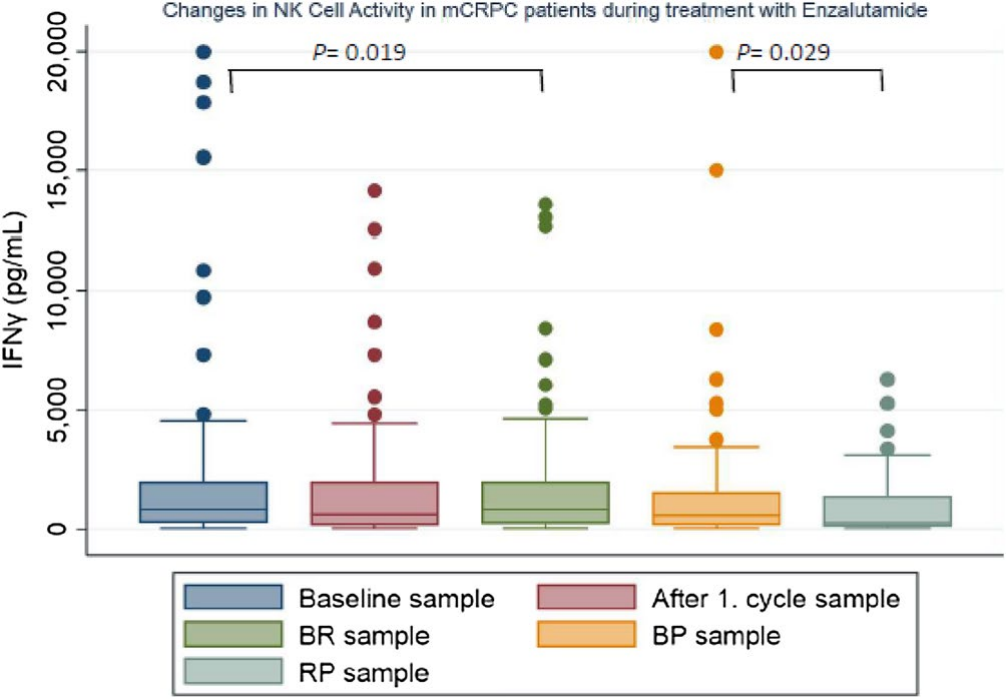
Boxplots illustrating changes in IFNγ in mCRPC patients during treatment with enzalutamide. *BR* biochemical response, *BP* biochemical progression, *RP* radiological progression.

There was a significant difference between patients in group 2 and patients in the other three groups regarding both OS, rPFS and TTT (HR 5.02, p = 0.003, HR 6.83, p =  < 0.001 and HR 7.05, p =  < 0.001 compared to group 1, respectively) (Fig. 2).

After adjustment for ISUP grade, baseline neutrophil count and NLR, there was still a significant shorter time to both rPFS and TTT observed in patients in group 2 compared to patients in group 1 (HR 4.30, p = 0.037; and HR 6.82, p = 0.011, respectively).

Tables 3 and 4 show the results of the both univariate and adjusted Cox regression analysis, respectively.

**Table 3:** Association between change in IFNγ plasma level and clinical outcomes in the four subgroups:

Groups	OS		rPFS		TTT	
	HR (95% CI)	P value	HR (95% CI)	P value	HR (95% CI)	P value
1	1		1		1	
2	5.02 (1.70–14.82)	**0.003**	6.83 (2.62–17.77)	** < 0.001**	7.05 (2.69–18.49)	** < 0.001**
3	1.33 (0.48–3.73)	0.581	1.77 (0.74–4.22)	0.196	1.46 (0.58–3.68)	0.378
4	1.12 (0.46–2.71)	0.798	1.79 (0.89–3.63)	0.101	1.54 (0.76–3.13)	0.199

**Table 4:** Association between change in IFNγ plasma level and clinical outcomes in the four subgroups after adjustment for baseline ISUP, baseline neutrophil count, and NLR:

Groups	OS		rPFS		TTT	
	HR (95% CI)	P value	HR (95% CI)	P value	HR (95% CI)	P value
1	1		1		1	
2	2.78 (0.57–13.59)	0.206	4.30 (1.09–16.93)	**0.037**	6.82 (1.55–30.03)	**0.011**
3	1.09 (0.34–3.50)	0.891	1.49 (0.55–3.99)	0.432	1.13 (0.39–3.26)	0.816
4	1.24 (0.35–4.47)	0.739	1.58 (0.63–3.94)	0.330	1.53 (0.59–3.92)	0.380

**Figure 3:**
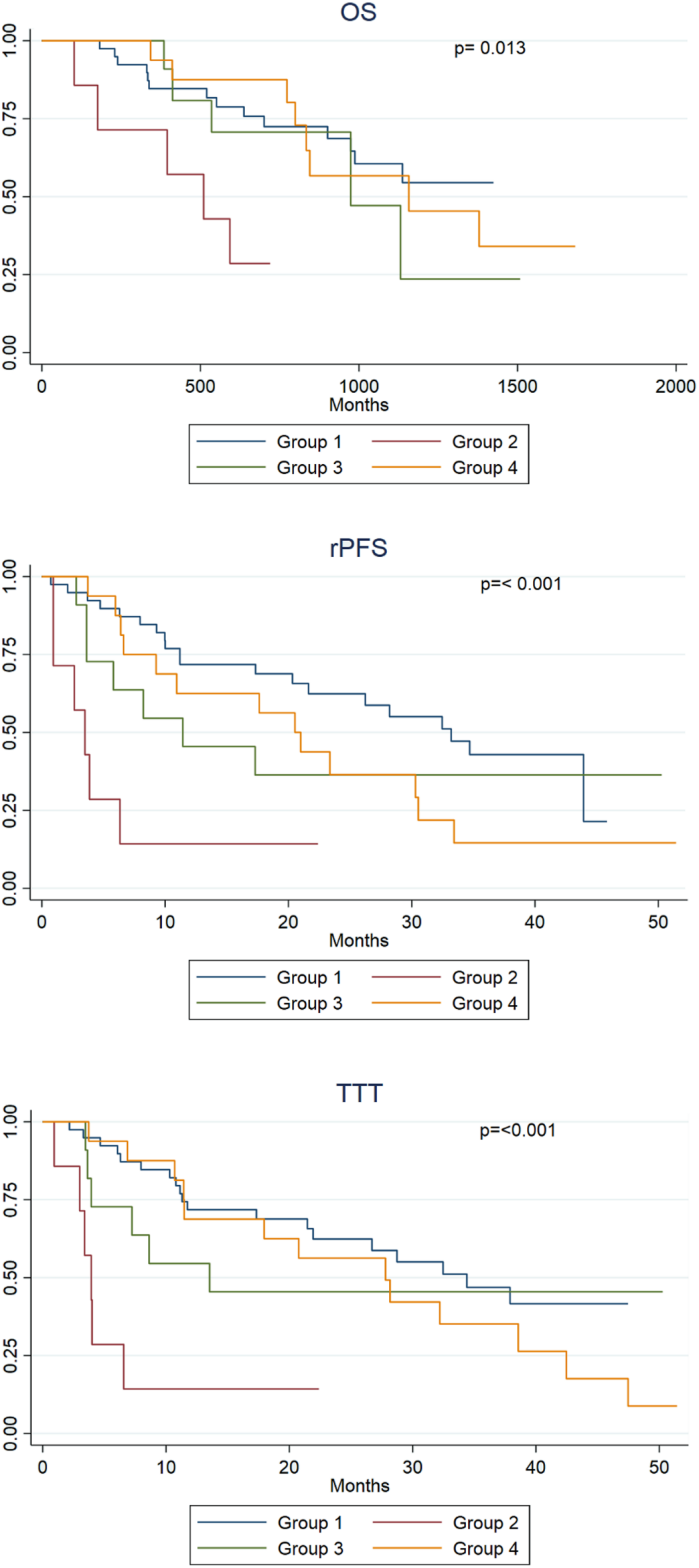
Kaplan–Meier overall survival (OS), radiological progression-free survival (rPFS), and time to new treatment (TTT) curves in the different groups treated by enzalutamide. Group 1 (n = 42) plasma level of IFNγ remained within a normal range (> 250 pg/mL), group 2 (n = 7), levels of IFNγ increased from an abnormal level at baseline (< 250 pg/mL) to normal values, group 3 (n = 13) levels of plasma IFNγ dropped to an abnormal level, and group 4 (n = 17) plasma level of IFNγ remained at an abnormal level. P values were obtained using log-rank test.

Relevantly, equivalent results were obtained when employing two other cut-offs from earlier studies on PCa patients (151 pg/mL^23^ and 500 pg/mL^24^). Both univariate and multivariate analyses of the outcomes data for those cutoffs are summarized in Tables S2 and S3 (Fig. 3).

Statistically significant values are in bold writing.

*OS* overall survival, *rPFS* radiological progression free survival, *TTT* time to new treatment.

Statistically significant values are in bold writing.

*OS* overall survival, *rPFS* radiological progression free survival, *TTT* time to new treatment.

### Association between NKA and diagnostic clinical characteristics:

Patients with baseline plasma levels of IFNγ ≥ 250 pg/mL (n = 53) had significantly lower values of baseline lactate dehydrogenase, and a tendency to higher levels of baseline basic phosphatase (z = − 2.25, p = 0.025 and z = 1.73, p = 0.084, respectively). Moreover, significantly higher ISUP scores were observed in patients with baseline IFNγ ≥ 250 pg/mL compared with those with baseline IFNγ < 250 pg/mL.

However, these two groups did not differ in terms of baseline PSA, baseline performance status or metastatic site at mCRPC stage (Table 5).

**Table 5:** Association between baseline IFNγ (≥ 250 pg/mL IFNγ and < 250 pg/mL IFNγ) and baseline clinical characteristics.

	PSA	BP	LDH	ISUP grade	Mets site	PS
z-score	− 1.26	1.73	-− 2.25	2.44	0.32	− 1.56
p-value	0.21	0.08	**0.03**	**0.02**	0.69	0.15

Statistically significant values are in bold writing.

*BP* basic phosphatase, ISUP: International Society of Urological Pathology, *LDH* lactate dehydrogenase, *PS* performance status, *PSA* prostate specific antigen, *Mets* metastases.

## Discussion

The role of NK cells and their activities have previously been explored in many types of cancers both as a diagnostic, predictive and prognostic marker. In PC it has been shown that both activity and antigen levels on NK cells may provide useful information in different stages of PC, however corresponding analyses in the late stage of the disease, namely mCRPC are lacking. For this reason, the present study investigated NKA in patients who were treated with enzalutamide as the first line of treatment in mCRPC.

In this study, level of IFNγ, measured as the surrogate marker of NKA in the employed assay, was significantly decreased at the time of BR compared to levels detected at baseline. Additionally, the level of IFNγ was significantly lower at RP compared to values at BP. Furthermore, an increase in IFNγ during the first three cycles of enzalutamide treatment was inversely correlated to both OS, rPFS and TTT. Finally, patients with an IFNγ plasma level below 250 pg/mL had significantly lower ISUP grade.

Changes in NKA during the treatment of patients with cancer seems to be highly dependent on many factors including; type of intervention (surgery, RT or medical treatment), intensity of treatment modality (mono- vs. combination therapy), and most importantly both cancer type- and stage (early vs. late stage).

In general, chemotherapies, with few exceptions tend to suppress not only the cytotoxic ability of NK cells, but also their cytokine productions^25^. Similarly, surgery in cancer patients including PC tends to reduce NKA^26,27^, while contradictory results have been reported regarding the impact of RT on NKA^28,29^.

In PC, castration treatment and antiandrogen do not seem to have a notable impact on NKA. Reportedly, in patients with mCSPC, NKA in peripheral blood was not affected by neither surgical castration nor antiandrogen (flutamide)^30^. Moreover, medical castration (with either agonist or antagonist) did not affect the intra-tumor CD56 + NK cells in tissue specimens from patients with local PC treated by prostatectomy compared to controls^31^.

Results from the present study demonstrated a significant decrease in IFNγ at BR compared to IFNγ levels measured before initiation of enzalutamide treatment. This observation contrasts the work by Lin et al., who showed that the sensitivity to enzalutamide treatment seemed to be improved by recruiting NK cells to CRPC cell line^32^. However, while our study analyzed peripheral blood of patients, the work by Lin and colleagues was an in vitro setting, therefore, it is debatable to what extend these two studies are comparable.

While the androgen receptor still plays a crucial role in the development of castration resistance, changes in immune cells were also observed to have a distinctive action during transmission of PC disease to mCRPC^33^. It’s well known that PC and especially the late stage of the disease, CRPC, have a very complex immunosuppressive tumor microenvironment^34^. Consequently, the role of NK cells in PC may differ from other cancer types. Additionally, the cytotoxic effect of NK cells could have a more important role for treatment response at the late stage of PCa than the effect of cytokines including IFNγ.

Finally, comparison of observations from other studies with the results in this work should be made with caution, as all patients in our study were castrated alongside treatment with enzalutamide. Moreover, differences in patient population may also be one of the reasons for such discrepancies, as all patients in abovementioned clinical studies were at CSPC stage.

The decrease in IFNγ at RP compared to BP observed in this study was quite expected, as the lag time from the disease at mCRPC stage progressed biochemically to there is a confirmed progression radiologically is well documented. In this cohort, the median time for both BP and RP was 7 months and 20 months, respectively, which is in parallel with those from both the PREVAIL^7^ study and the RWD studies^35^.

Regarding outcomes, the present study showed that mCRPC patients in group 2 (those who went from a low IFNγ level at baseline to a normal level during the first three cycles of enzalutamide) had shorter OS, rPFS, and TTT than patients in other groups. Furthermore, neither patients with consistent low levels or decreasing levels of IFNγ differed from patients having normal levels during the follow-up period in terms of OS, rPFS and TTT.

Despite the differences between these four groups in some of their baseline clinical characteristics and in baseline plasma IFNγ levels, patients in group 2 still showed a significantly shorter time to both, rPFS and TTT after multivariate adjustment. However, the interpretation of these observations should be carried out carefully owing to the relative small size of group 2 (only 7 patients).

Chowdhury et al.^36^ observed a decrease in circulating CD56^bright^ in mCRPC patients treated with dendritic-tumor cell hybridom vaccine (aHyC vaccine), although the beneficial effect on patients’ survival. Such changes in NK cell subsets may instigate alterations in levels of regulatory cytokines and chemokines as we have observed in the present study. On the other hand, in a phase IIa study, higher levels of IFNγ were detected in mCRPC patients with no RP, compared to those with progressive disease after being vaccinated by dendritic cells (DC)^37^.However, two main differences between these two trials that could explain such discrepancies. Firstly, mCRPC population in DC vaccine trial was exposed to different treatment regimens before recruiting in the trial; such as enzalutamide, apalutamide, and abiraterone. Secondly, IFNγ measured in DC vaccine trial was produced by tissue-infiltrating T helper 1 cell and not by circulating NK cells as we are measuring.

Importantly, in line with our results, in hematological malignancies such as malignant melanoma^38^ and acute lymphoblastic leukemia^39^, an inverse correlation between NKA and outcomes has also observed.

The positive prognostic value of high NKA in local settings of PC has previously been documented^23,40^. Likewise, in metastatic PC, Pasero et al. investigated NKA in blood samples from 39 patients with de novo mCSPC, and observed a positive correlation between high expression of NK activating receptors and cytotoxicity with both time to CRPC and OS^41^.

Moreover, our group has previously documented a significant shorter PFS in cancer patients with decreasing plasma levels of IFNγ within the first two months of treatment. However, it’s worth noting that, data from that work were pooled from patients with different types of cancer where mCRPC patients represented only 20% of the whole population^42^.

Additionally, the poor prognostic value of high neutrophil to lymphocyte ratio (NLR) is well documents in manty cancers including mCRPC^43^Eventhough NLR has also a negative impact on outcomes for patients included in the current study, patients in group 2 still had significant shorter duration both to rPFS and the new treatment compared to patients in all the three groups, even after adjustment for the effect of both NLR and baseline neutrophil.

It is quite challenging to explain and account for the effect that NK cells may have in mCRPC, and as IFNγ was the only marker investigated in the present work every supposition will be purely speculative.

This inverse correlation between IFNγ levels and outcome in some studies on cancer patients could be due to the paradoxical effect of NK cells on regulatory T cells in late stage of cancer diseases through both increased expression of CD38 and higher production of IFNγ^38^.

Another possible explanation is that hyper activated NK cells could lead to both dysfunctionality and inability to lyse NK cell sensitive targets^39^. This has been observed by Santos et al. while exploring the cytotoxic activity of circulating NK cells derived from 74 cancer patients (including eight patients with mPC). They reported a significant decrease in NK cell cytotoxic activity (by the ability to lyse K562 cell line), despite the marked increase in NK cell number^44^.

Additionally, it was hypothesized that such increase in NK cytokines could be a compensatory mechanism for the reduced CD57 expression , a marker for NK maturity due to effect of ADT^45^.

Understanding the complexity of the role of NK cells in mCRPC is still in its infancy, and therefore, such inconsistency in aforementioned observations is anticipated, and could be explained by integration of many factors.

The use of pre-defined cut-off in the translational studies can be always questioned. As stated earlier, the selected cut-off for plasma level of IFNγ in this study (250 pg/ml) is pre-defined by the manufacturer. Importantly, this value is in the same range as cut-offs calculated and reported by others that have used the NK Vue® assay in previous investigations on different types of cancer including PCa^46–48^. However, this study is the first one to employ this pre-defined cut-off on patients diagnosed with mCRPC. To validate the current findings, two other cut-offs obtained from earlier studies on PCa patients (151^23^ and 500 pg/ml^24^) were also tested for outcomes data on the current cohort. Using these, we still found that patients in group 2 showed a shorter time to both rPFS and TTT, thus indicating that 250 pg/ml IFNγ is in fact a relevant cut-off for studying mCRPC.

The natural biological and immunological differences between different malignant diseases, possible heterogeneity among both populations and stages within the same cancer, different treatment options used, and the variety of NKA markers investigated in these studies can easily contribute to such conflicting conclusions. The paradoxical effect of both innate and adaptive immune responses has been reported in different type of cancers^49^. For instance; the IFNγ-mediated anti-tumor role is well described, however, recent observation from preclinical studies questioned such role. Hence, in recent investigations there are clear indications that IFNγ also has pro-tumorigenic effects allegedly by promoting epithelial-to mesenchymal transition in both renal cancer^50^, and PC^51^, and by inducing stemness in tumor microenvironment in a dose dependent manner in lung cancer^52^.

There have been limitations for this study; there is a lack of information about corticosteroid replacement therapy during the study, patients with autoimmune disease were not excluded and only a modest number of patients were included. Additionally, there was heterogeneity in baseline diagnostic characteristics as half of the patients had metastatic disease already at the time of diagnosis. Lastly, introduction of chemotherapy in up-front setting in this study could also have an influence on NKA later on in the mCRPC stage^53^. This study could have encompassed C-Reactive Protein (CRP) as this acute phase reactant is also prognostic in PC, but more importantly, because it is not known if the level of CRP associates with and/or impacts NKA. Unfortunately, CRP was not measured in this cohort^54^.

## Conclusion

The inverse correlation between level of IFNγ and both treatment response, outcomes, and prostate tumor differentiation in mCRPC patients in this study may suggest an adverse effect of NK cells at that stage. Validation studies exploring NKA on larger cohorts with different treatment options are required for a better interpretation of these observations.

### Supplementary Information

Supplementary Figure S1.

Supplementary Figure S2.

Supplementary Table S1.

Supplementary Table S2.

## Data Availability

The datasets generated during and/or analysed during the current study are available from the corresponding author on reasonable request.
