# Supplementary material for: Natural killer cell activity in metastatic castration resistant prostate cancer patients treated with enzalutamide
Source: Sci Rep. 2023 Oct 10;13:17144. doi: 10.1038/s41598-023-43937-7 (PMC10564750; doi:10.1038/s41598-023-43937-7)
Supplement: Supplementary file 2 — Supplementary Figure S2. [file 41598_2023_43937_MOESM2_ESM.docx]

Figure S2: Box plots showing the changes in IFNγ plasma level during the first 3 cycles of enzalutamide treatment for each group. Group 1 (n = 42) plasma level of IFNγ remained within a normal range (≥ 250 pg/mL), group 2 (n = 7), levels of IFNγ increased from an abnormal level at baseline (< 250 pg/mL) to normal values, group 3 (n = 13) levels of plasma IFNγ dropped to an abnormal level, and group 4 (n = 17) plasma level of IFNγ remained at an abnormal level.
