# Supplementary material for: Natural killer cell activity in metastatic castration resistant prostate cancer patients treated with enzalutamide
Source: Sci Rep. 2023 Oct 10;13:17144. doi: 10.1038/s41598-023-43937-7 (PMC10564750; doi:10.1038/s41598-023-43937-7)
Supplement: Supplementary file 3 — Supplementary Table S1. [file 41598_2023_43937_MOESM3_ESM.docx]

**Table S1: Baseline clinic-pathological characteristics for the four subgroups**

|  | Group 1 N= 42 | Group 2 N= 7 | Group 3 N=13 | Group 4 N=17 | *All groups*  *p-value* | *Group 2 vs. rest the cohort*  *p-value* |
| --- | --- | --- | --- | --- | --- | --- |
| Mean age at Dx, years  (range) | 70  (51-83) | 74  (71-80) | 69  (52-81) | 71  (59-78) | 0.23 | 0.13 |
| Mean age at inclusion, years  (range) | 75  (61-89) | 77  (72-85) | 74  (54-86) | 76  (66-86) | 0.80 | 0.74 |
| Median PSA level at Dx, ng/mL (range) | 45  (2-6000) | 167  (6-2200) | 232  (10-5000) | 52  (6-600) | 0.40 | 0.74 |
| Median baseline PSA level, ng/mL (range) | 26  (1-223) | 98  (6-138) | 47  (3-238) | 41  (2-396) | 0.66 | 0.18 |
| Median baseline neutrophils level, 10^9/L (range) | 3.89  (1.85-8.25) | 6.54  (4.74-8.98) | 4.51  (2.83-7.83) | 5.49  (2.4-8.95) | <0.01 | <0.01 |
| Median baseline NLR (range) | 2,49  (0,96-6,53) | 4,21  (2,53-13,83) | 3,72  (1,56-4,68) | 3,28  (2,13-15,40) | 0.03 | 0.04 |
| Median baseline BP level, ng/mL (range) | 92  (39-394) | 105  (60-313) | 116  (11-658) | 81  (43-1983) | 0.18 | 0.41 |
| Median baseline LDH level, ng/mL (range) | 198  (118-291) | 236  (187-283) | 180  (143-286) | 207  (156-325) | 0.35 | 0.05 |
| Median baseline IFNγ level, pg/mL (range) | 1914  (282-20000) | 195  (32-245) | 564  (255-1994) | 32  (32-133) | **<0.01** | **0.13** |
| Baseline PS  0 1 2 N/A | 25 10 1 6 | 3 3 1 0 | 8 3 1 1 | 8 5 1 3 | 0.80 | 0.20 |
| Baseline ISUP  I II III IV V N/A | 1 3 8 10 19 1 | 2 0 0 3 2 0 | 1 0 3 1 7 1 | 3 4 6 2 2 0 | **0.01** | **0.69** |
| *De novo* mPCa (%) | 17 (40%) | 4 (57%) | 9 (69%) | 6 (35%) | 0.22 | 0.52 |
| Previous treatment (%)⃰  RARP RT DOC up-front | 14 (33)  3 7 5 | 1 (14%)  0 0 1 | 10 (77%)  2 1 7 | 4 (24%)  2 2 1 | 0.41 |  |
| Mets sites^∆^  LN Bone Visceral mets. | 17 23 3 | 3 5 0 | 8 13 0 | 8 13 2 | **0.024** | **0.58** |
| Time to mCRPC, mo, median (range) | 48  (10-227) | 21  (18-103) | 30  (8-238) | 60  (13-175) | 0.28 | 0.06 |
| Time from initial management to 1. Cycle Enzalutamide, mo, median (range) | 57  (6-193) | 18 | 23  (4-231) | 99  (19-168) | 0.19 | 0.47 |
| Patients exposed for dose reduction, n (%)  Yes  No | 18 (43)  24 (57) | 3 (43)  4 (57) | 2 (15)  11 (85) | 5 (29)  12 (71) |  | 0.67 |
| Synchronic cancer (%) | 1 | 0 | 0 | 1 |  |  |
| Previous Cancer (%) | 4 | 0 | 0 | 2 |  |  |
| Auto-immune disease (%) | 1 | 1 | 1 | 2 |  |  |

ADT: anti-deprivation therapy; BP: Basic phosphatase; DOC: Docetaxel; Dx: Diagnosis; LN: Lymph nodes; IFNγ; Interferon gamma; ISUP: International Society of. Urological Pathology; LDH: Lactate dehydrogenase; mCRPC: metastatic castration resistant prostate cancer; mo: month; PSA: prostate specific antigen; PS: performance status; RARP: robot assisted radical prostatectomy; RP: radiological progression; RT: radiotherapy.

⃰: some patients have received more than one previous treatment before included in this study.

∆: some patients have metastases to more than one location (eg. to both LN and bone)
