# Supplementary material for: Natural killer cell activity in metastatic castration resistant prostate cancer patients treated with enzalutamide
Source: Sci Rep. 2023 Oct 10;13:17144. doi: 10.1038/s41598-023-43937-7 (PMC10564750; doi:10.1038/s41598-023-43937-7)
Supplement: Supplementary file 4 — Supplementary Table S2. [file 41598_2023_43937_MOESM4_ESM.docx]

| \|  \|  \| 250 pg/mL \| \| \| 151 pg/mL \| \| \| 500 pg/mL \| \| \| \| --- \| --- \| --- \| --- \| --- \| --- \| --- \| --- \| --- \| --- \| --- \| \| Groups \| HR (95% CI) \| P value \| Log rank \| HR (95% CI) \| P value \| Log rank \| HR (95% CI) \| P value \| Log rank \| \| OS \| 1 \| 1 \|  \| **0.013** \| 1 \|  \| 0.453 \| 1 \|  \| 0.078 \| \| 2 \| 5.02 (1.70-14.82) \| **0.003** \| 2.56 (0.73-8.93) \| 0.140 \| 4.32 (133-14.00) \| **0.015** \| \| 3 \| 1.33 (0.48-3.73) \| 0.581 \| 1.54 (0.56-4.22) \| 0.400 \| 1.32 (0.29-5.90) \| 0.718 \| \| 4 \| 1.12 (0.46-2.71) \| 0.798 \| 1.23 (0.52-2.90) \| 0.631 \| 133 (0.61-2.90) \| 0.472 \| \|  \|  \|  \|  \|  \|  \|  \|  \|  \|  \|  \| \| rPFS \| 1 \| 1 \|  \| **<0.001** \| 1 \|  \| **0.049** \| 1 \|  \| **0.009** \| \| 2 \| 6.83 (2.62- 17.77) \| **< 0.001** \| 3.90 (1.33-11.41) \| **0.013** \| 5.06 (1.82-14.04) \| **0.002** \| \| 3 \| 1.77 (0.74- 4.22) \| 0.196 \| 1.69 (0.69-4.16) \| 0.253 \| 1.31 (0.38-4.41) \| 0.664 \| \| 4 \| 1.79 (0.89- 3.63) \| 0.101 \| 1.60 (0.80-3.21) \| 0.185 \|  \|  \| \|  \|  \|  \|  \|  \|  \|  \|  \|  \|  \|  \| \| TTT \| 1 \| 1 \|  \| **<0.001** \| 1 \|  \| 0.074 \| 1 \|  \| **0.010** \| \| 2 \| 7.05 (2.69-18.49) \| **<0.001** \| 3.86 (1.32-11.34) \| **0.014** \| 4.88 (1.75-13.61) \| **0.002** \| \| 3 \| 1.46 (0.58-3.68) \| 0.378 \| 1.31 (0.50-3.47) \| 0.586 \| 0.89 (0.21-3.83) \| 0.872 \| \| 4 \| 1.54 (0.76-3.13) \| 0.199 \| 1.36 (0.67-2.74) \| 0.396 \| 1.46 (0.76-2.79) \| 0.256 \| |
| --- | --- | --- | --- | --- | --- | --- | --- | --- | --- | --- | --- | --- | --- | --- | --- | --- | --- | --- | --- | --- | --- | --- | --- | --- | --- | --- | --- | --- | --- | --- | --- | --- | --- | --- | --- | --- | --- | --- | --- | --- | --- | --- | --- | --- | --- | --- | --- | --- | --- | --- | --- | --- | --- | --- | --- | --- | --- | --- | --- | --- | --- | --- | --- | --- | --- | --- | --- | --- | --- | --- | --- | --- | --- | --- | --- | --- | --- | --- | --- | --- | --- | --- | --- | --- | --- | --- | --- | --- | --- | --- | --- | --- | --- | --- | --- | --- | --- | --- | --- | --- | --- | --- | --- | --- | --- | --- | --- | --- | --- | --- | --- | --- | --- | --- | --- | --- | --- | --- | --- | --- | --- | --- | --- | --- | --- | --- | --- | --- | --- | --- | --- | --- | --- | --- | --- | --- | --- | --- | --- |

Table S2: Association between change in IFNγ plasma level and clinical outcomes in the four subgroups with all three different cut-offs:

OS: Overall survival; rPFS: radiological progression free survival; TTT: time to new treatment. Statistically significant values are in bold writing.

|  |  | 250 pg/mL | | 151 pg/mL | | 500 pg/mL | |
| --- | --- | --- | --- | --- | --- | --- | --- |
|  | Groups | HR (95% CI) | P value | HR (95% CI) | P value | HR (95% CI) | P value |
| OS | 1 | 1 |  | 1 |  | 1 |  |
|  | 2 | 3.86 (0.91-16.43) | 0.067 | 1.95 (0.30-12.58) | 0.480 | 3.89 (0.89-17.00) | 0.071 |
|  | 3 | 1.64 (0.47- 5.68) | 0.435 | 2.54 (0.76-8.52) | 0.130 | 1.47 (0.30-7.19) | 0.638 |
|  | 4 | 0.90 (0.23- 3.49) | 0.881 | 1.52 (0.44-5.25) | 0.508 | 1.41 (0.42-4.65) | 0.577 |
|  |  |  |  |  |  |  |  |
| rPFS | 1 | 1 |  | 1 |  | 1 |  |
|  | 2 | 5.50 (1.63-18.58) | **0.006** | 3.02(0.67-13.72) | 0.148 | 4.81 (1.37-16.88) | **0.014** |
|  | 3 | 2.47 (0.85- 7.17) | 0.097 | 2.74 (0.87-8.68) | 0.087 | 1.18 (0.33-4.26) | 0.798 |
|  | 4 | 1.64 (0.60- 4.51) | 0.335 | 1.77 (0.66-4.73) | 0.254 | 1.47 (0.55-3.91) | 0.438 |
|  |  |  |  |  |  |  |  |
| TTT | 1 | 1 |  | 1 |  | 1 |  |
|  | 2 | 6.38 (1.87-21.81) | **0.003** | 3.40 (0.72-16.03) | 0.122 | 4.97 (1.39-17.76) | **0.014** |
|  | 3 | 1.89 (0.61- 5.88) | 0.271 | 1.86 (0.54-6.42) | 0.328 | 0.87 (0.19-4.04) | 0.864 |
|  | 4 | 1.61 (0.58- 4.48) | 0.366 | 1.68 (0.63-4.48) | 0.303 | 1.59 (0.59-4.31) | 0.359 |

Table S3: Association between change in IFNγ plasma level and clinical outcomes in the four subgroups with all three different cut-offs after adjustment for baseline clinical characteristics:

OS: Overall survival; rPFS: radiological progression free survival; TTT: time to new treatment. Statistically significant values are in bold writing.
